# VEGF is an important mediator of tumor angiogenesis in malignant lesions in a genetically engineered mouse model of lung adenocarcinoma

**DOI:** 10.1186/1471-2407-13-213

**Published:** 2013-04-29

**Authors:** Bharat K Majeti, Joseph H Lee, Brett H Simmons, Farbod Shojaei

**Affiliations:** 1 Oncology Research Unit, Pfizer Inc, La Jolla, CA, 92121, USA

**Keywords:** Lung cancer, Kinase and phosphatase inhibitors, Tumor angiogenesis, GEMM, VEGF

## Abstract

**Background:**

VEGF is one of the key drivers of physiological or pathological angiogenesis hence several VEGF inhibitors are in different stages of clinical development. To further dissect the role of VEGF in different stages of tumor progression in lung tumors, we utilized Kras^G12D-LSL^ GEMMs (genetically engineered mouse models).

**Methods:**

Intranasal delivery of adenoviruses expressing cre recombinase in Kras^G12D-LSL^ mice results in the expression of mutant Kras that leads to development of tumor lesions ranging from adenomatous hyperplasia to large adenoma and adenocarcinoma over time in lung. In the current study, we treated Kras^G12D-LSL^ mice at 14 weeks post inhalation with three different angiogenic inhibitors including axitinib and PF-00337210 both of which are selective inhibitors of VEGFR and sunitinib which targets VEGFR, C-SF1-R, PDGFR and KIT.

**Results:**

Pathology findings showed no significant difference in percentage of adenomatous hyperplastic lesions between the vehicle *vs.* any of the treatments suggesting that angiogenesis may not play a major role at early stages of tumorigenesis. However, each inhibitor suppressed percentage of benign adenoma lesions and almost fully inhibited growth of adenocarcinoma lesions in the recipients which was consistent with a reduction in tumor vasculature. Treatment with sunitinib which is a multi-targeted RTKI did not provide any advantage compared to selective VEGFR inhibitor further emphasizing role of VEGF in tumor angiogenesis in this model.

**Conclusion:**

Overall, our studies indicate significance of VEGF and angiogenesis in a spontaneous model of lung tumorigenesis and provide a proof of mechanism for anti-cancer activity of VEGF inhibitors in this model.

## Background

Angiogenesis, formation of new blood vessels from existing vasculature, is an important process that supplies required nutrients and oxygen to cells which are distant from existing blood vessels. Angiogenesis is proven to play a key role in tumor growth and progression and several angiogenic factors such as VEGF (vascular endothelial growth factor), PDGF (platelet derived growth factor), bFGF (basic fibroblast growth factor) and HGF (hepatocyte growth factor) found to be among key regulators of tumor angiogenesis [1].

Series of investigations demonstrate a key role for VEGF in physiological or pathological angiogenesis [1]. Hence, a number of anti-angiogenic drugs targeting VEGF signaling pathway (ligand or the receptors) have been developed and are currently in use in cancer therapy. Bevacizumab (an anti-VEGF monoclonal antibody) was the first angiogenic inhibitor (hereafter AI) initially approved for use in patients with NSCLC (non-small cell lung cancer) or mCRC (metastatic colorectal cancer) [2,3]. Small molecule inhibitors of receptor tyrosine kinase inhibitors (RTKIs) are another class of agent targeting VEGF signaling pathway. RTKIs such as sunitinib, sorafenib, cediranib, motesanib, pazopanib and axitinib have been approved or are being tested in different phases of clinical trials. Sunitinib which is a multi-targeted kinase inhibitor targets VEGFRs, C-SF1R, KIT and also platelet-derived growth factor (PDGFR) which plays an important role in blood vessel maturation [4]. Recently, sunitinib was approved by FDA for the treatment of advanced renal cell carcinoma, gastrointestinal stromal tumors and pancreatic neuroendocrine tumors [5,6]. Axitinib (AG-013736; Pfizer) is another oral potent tyrosine kinase inhibitor which mainly targets VEGFR and was approved by FDA for use in patients with advanced RCC [7]. In a murine lewis lung carcinoma model, single agent axitinib induced tumor necrosis and reduced microvessel density [8]. PF-00337210 (hereafter PF-210) is an oral, potent ATP-competitive inhibitor of VEGFR family [9]. It inhibits VEGFR2 phosphorylation and has greater selectivity towards VEGFR2 than other kinases. PF-210 has been shown to inhibit HUVEC cell survival *in vitro* and suppresses tumor angiogenesis in xenograft models [10].

Ras superfamily of proteins regulates cell growth, survival, and differentiation. Hras, Kras 4a, Kras 4b and Nras are the four highly homologous proteins encoded by three *Ras* genes [11,12]. Mutations in the *KRAS* gene lead to KRas protein activation in many human tumors including NSCLC, pancreatic cancer and colorectal cancer [12-14]. The majority of *KRAS* mutations (approximately 97%) occur in exon 2 at codon 12 and/or codon 13 in NSCLC patients [12]. The most common mutation in *KRAS* occurs at position 12, where glycine is replaced by a residue with side chain. NSCLC patients represent the majority of all lung cancer patients and remain a major cause of death [12]. Hence, Kras^G12D-LSL^ GEMM (genetically engineered mouse model) is one of the most relevant models of NSCLC to study tumor progression and to investigate efficacy of anti-cancer agents.

In the present study we investigated anti-tumor efficacy of three RTKIs including sunitinib, axitinib and PF-210 in Kras^G12D-LSL^ lung tumor model. Irrespective of the type (multi-targeted or selective), all three inhibitors significantly inhibited growth of advanced (adenocarcinoma) lesions in the lung indicating that VEGF is a key regulator of tumor angiogenesis in this model.

## Methods

### Tumor development and treatment in Kras^G12D-LSL^ GEMMs

Kras^G12D-LSL^ heterozygous mice were obtained from Jackson Laboratories (Jax West, CA) at approximately 3–4 weeks of age and were maintained by Pfizer La Jolla comparative medicine under guidelines provided by IACUC (Institutional Animal Care and Use Committee). Lung tumors were generated in Kras^G12D-LSL^ mice, using a recently published protocol [15]. Briefly, adenovirus expressing Cre recombinase (Adeno-Cre;the University of Iowa Gene Transfer Vector Core, Iow, IA) were titrated by Adenoviral Titration Kit (Clontech, CA) using instruction provided by the manufacturer. Prior to administration, Adeno-Cre virus was prepared in 50 ul of plain MEM (minimal essential media; Gibco BRL; life Sciences, CA) supplemented with CaCl2 (10 mM) followed by incubation at room temperature (RT) for 20 minutes. The recipients (n = 10) were anesthetized using Ketamine (Baxter) and Xylazine (Vedco) and the adeno-Cre preparation (2.5 × 10^7 infectious units; IU) was administered intra-nasally. To monitor tumor formation and progression, lung tissue was isolated (n = 1-3) at several time points (4, 8 and 12 wks) post inhalation and were stained with H&E (Hematoxylin and Eosin) using standard protocols in the laboratory [15]. The inhaled mice were randomized at 14 wks post-inhalation and were treated with vehicle, sunitinib (40 mg/kg qd), axitinib (15 mg/kg bid) and PF-210 (40 mg/kg qd) using oral route of administration and formulation protocols as described previously [8]. All the animal study procedures were monitored by the veterinary personnel to comply with guidelines provided by IACUC.

To assess therapeutic response to angiogenic inhibitors, lung lesions were quantified in the recipients by a certified pathologist. As previously described, lesions were categorized as hyperplastic, benign adenoma and adenocarcinoma [15]. Lesion quantification provided two types of analyses in the recipients: 1) percentage of each type of lesion in the recipient lung; 2) percentage of mice carrying these lesions in each treatment. To provide statistical analyses, we applied student’s *t* test (p < 0.05 considered significant) to compare data between the vehicle *vs.* each treatment.

### Histology

Formalin fixed paraffin embedded lung tissues were cut into 5 μm sections and were stained for CD31, desmin, and F4-80 separately. Immunohistochemical staining was performed on Leica Bond III automated machine. Bond polymer refine detection kit was used for desmin and CD31 staining and bond intense R detection was used for F4-80 staining. For CD31 staining, lung sections were incubated for 45 minutes with rabbit anti-CD31 monoclonal antibody (clone SP38, Spring Bioscience, cat # M3384, 1:100 dilution). Desmin was stained by incubating lung section with mouse anti-huDesmin antibody (Dako Cytomation, cat# M0760, 1:1500 dilution) for 15 minutes. VEGFR1 and VEGFR2 was stained using anti-VEGFR1 antibody (abcam, cat# ab2350, 1:400 dilution) and anti-VEGFR2 antibody (cell signaling, cat# 2479, 1:200 dilution) respectively. Finally, F4-80 was stained with biotin anti-mouse F4-80 antibody (eBioscience, Cat # 13-4801-82, 1:75 dilution and 45 minutes incubation at RT). Images of stained-slides were captured using a Nanozoomer instrument (Hamamatsu, Japan) and the data was analyzed using Aperio Imagescope software.

## Results

### Targeting the VEGF pathway is sufficient to inhibit progression of lung adenocarcinoma lesions in Kras^G12D-LSL^ mice

**Figure 1:**
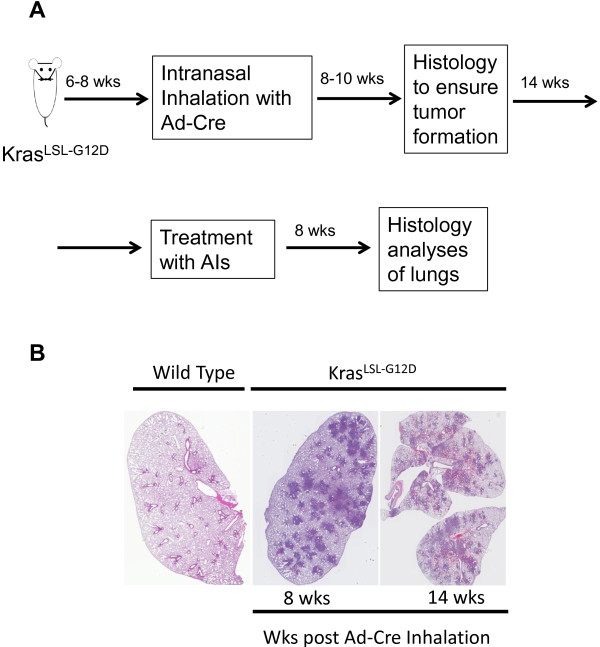
**Strategy to investigate efficacy of AIs in lung tumors in Kras**^**G12D-LSL**^**. A** To investigate efficacy of AIs in lung tumors in Kras^G12D-LSL^, mice were intranasally inhaled with Adeno-Cre and monitored at 8 wks after inhalation by histology analyses of few lungs from the recipients. All the remaining mice were randomized into groups for treatment with AIs (PF-210, axitinib and sunitinib) at 14 wks after Adeno-Cre administration when majority of animals develop malignant lesions in the lung. At 8 weeks after treatment, all the lungs from the treated mice were histologically analyzed. **B** Representative Images of lung lesions at 8 and 14 wks after Adeno-Cre administration compared to a lung isolated from a wild type mouse.

**Figure 2:**
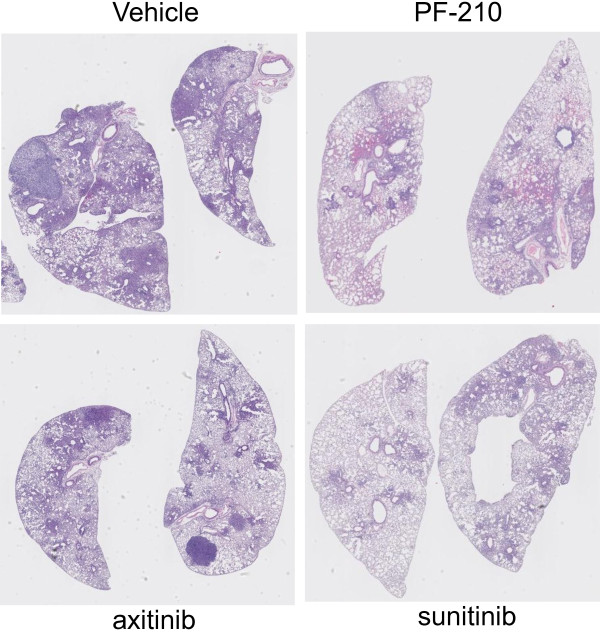
**AIs suppress growth of advanced lesions in lung tumors in Kras**^**G12D-LSL **^**mice.** As described in Figure 1, Kras^G12D-LSL^ tumor bearing mice were treated for 8 wks with AIs. Treatments started at 14 wks post administration of Adeno-Cre. At the end of study, all the lung tissues were isolated from the recipients and were analyzed using H&E staining. Images are the representative of each treatment.

**Figure 3:**
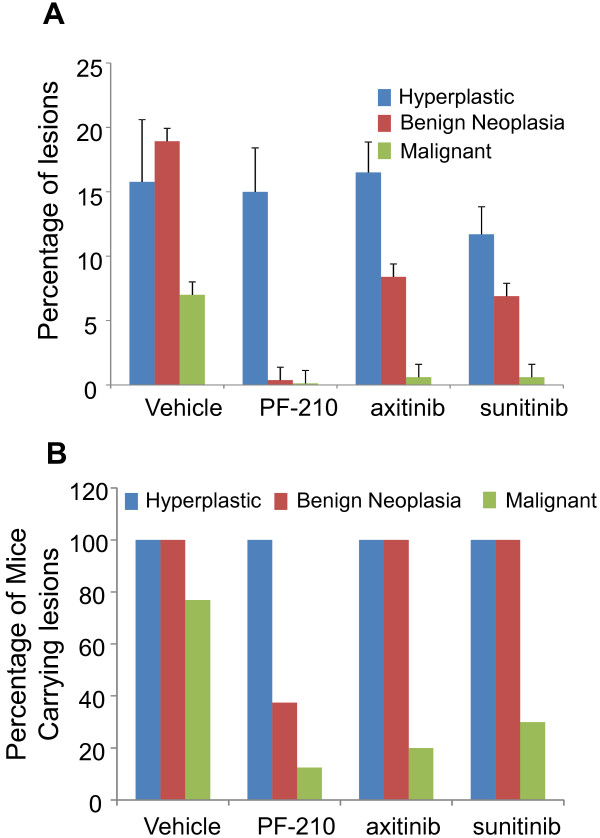
**Benign neoplasia and malignant lesions are targeted by AIs. A** lung lesions were divided into three groups including hyperplasia, benign neoplasia and malignant as described [15]. Hyperplastic and benign neoplasia comprise majority of lesions in this model followed by malignant lesions. While percentage of hyperplastic lesions has not significantly changed in any of the AIs treatment, while PF-210 treatment significantly reduced the percentage of benign andenoma lesions. Axitinib and sunitinib also targeted benign adenoma lesions. All the AIs consistently targeted percentage of malignant lesions in the lungs. **B** Analysis of percentage of mice carrying each lesion in each treatment was consistent with the above finding. All the treated mice carried hyperplasia and, with the exception of PF-210, benign neoplasia lesions. Percentage of mice carrying malignant lesions was significantly reduced in all the mice treated with AIs. Asterisks indicate significant difference (p < 0.05) when comparing lesions in AI treated mice vs. corresponding vehicle-treated animals.

Our strategy to investigate anti-tumor efficacy of AIs in Kras^G12D-LSL^ mice is depicted in Figure 1A. Kras^G12D-LSL^ mice were inhaled intranasally with Adeno-Cre at 6–8 weeks of age and were maintained without any further intervention. At 8–10 weeks post inhalation, few mice (n = 1-3) were randomly euthanized to assess tumor formation and progression in the lung (Figure 1B). All the remaining mice were randomized into treatment groups and treated with vehicle, PF-210, axitinib and sunitinib for about 8 weeks. Upon termination of the study, lung tissues were analyzed for tumor lesions using H&E staining (Figure 2). Compared to vehicle-treated group, there was a significant reduction in lung lesion in all the three drugs treated groups. To further understand mechanism of action of AIs, we classified lung lesions into three categories including hyperplasia, benign neoplasia and malignant (adenocarcinoma). Detailed pathology analyses of lesions revealed that hyperplastic lesions were not significantly affected by AIs compared to control-treated animals (Figure 3A). However, the percentage of benign neoplastic lesions was significantly (p < 0.01) inhibited by PF-210 (more than 90%) and also axitinib or sunitinib (more than 50%) compared to vehicle-treated mice. Finally malignant lesions were significantly (p < 0.01) inhibited by all the AIs. Additionally we investigated percentage of mice carrying the above-mentioned lesions (Figure 3B). Irrespective of the type of treatment, all mice carried hyperplastic lesions. While all mice treated with axitinib or sunitinib carried benign neoplasia, only 40% of PF-210 treated animals carried these lesions indicating the potency of this compound. Finally all three AIs reduced frequency of malignant lesions by at least 50% in treated mice (Figure 3B). Overall, two types of analyses (percentage of lesions in the lung and percentage of mice carrying specific lesion) indicate that AIs specifically target advanced lesions (malignant or adenocarcinoma).

### Components of vasculature and stroma are targeted by AIs

**Figure 4:**
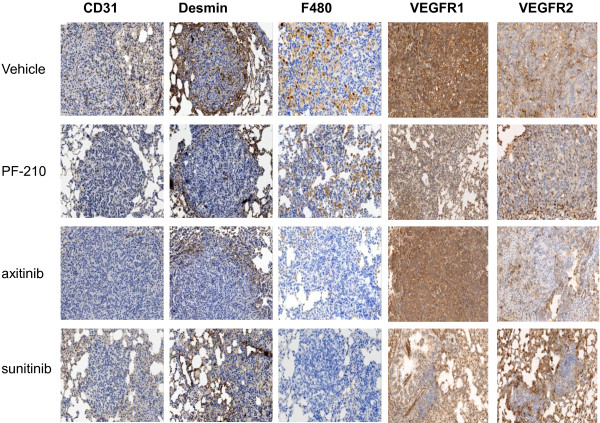
**Tumor vasculature and components of stroma in lung tumors are targeted by anti-angiogenesis.** Formalin fixed paraffin embedded lung sections (5 μm) stained with anti-CD31 (endothelial cells), anti-desmin (smooth muscle cells), anti-F4-80 (macrophages), anti-VEGFR1 and anti-VEGFR2 as described in the materials and methods. The slides were scanned on Nanozoomer instrument using 20× magnification. Irrespective of treatment, AIs treated lungs showed lower CD31 and desmin density. F4-80 staining in vehicle treated mice showed greater infiltration of macrophages. However, AIs particularly axitinib and sunitinib significantly reduced macrophage infiltration in the tumors. AIs had differential effects on VEGFR1 and VEGFR2 expression since all treatments reduced VEGFR2 expression in the tumor vasculature, VEGFR1 expression was mainly inhibited by PF-210 and sunitinib. Pictures are the representative images of each staining from each treatment.

To further investigate tumor vasculature, we stained lung tissues with different markers such as CD31 and desmin to stain endothelial cells and smooth muscle cells respectively [16]. Vasculature analysis by CD31 staining showed high density of tumor blood vessels in adenoma and adenomacarcinoma lesions in the vehicle group (Figure 4). Moreover, these vessels were desmin positive indicative of a mature vasculature in these lesions. In contrast, tumor lesions in AI treated groups had less number of blood vessels further suggesting that vasculature is the main target of these AIs. Additionally, vasculature was found to be more fragmented compared to the blood vessels in vehicle treated mice. Similar to CD31 staining, all three AIs (especially PF-210 and axitinib) targeted smooth muscle cells suggesting that not only blood vessels but also other components of vasculature are affected. . We also investigated the effects of AIs on the expression of VEGFR1 and VEGFR2 which play an important role in angiogenesis and tumor progression [1]. High levels of VEGFR1 was observed on tumor cells in vehicle treated mice (Figure 4) that is consistent with the expression of VEGFR1 on tumor cells isolated from Kras mutant NSCLC tumors in an earlier report [17]. Interestingly, sunitinib and PF-210, but not axitinib, inhibited VEGFR1 expression on tumor cells (Figure 4). Compared to vehicle treated tumors that expressed abundant levels of VEGFR2 on blood vessels, all three AIs inhibited VEGFR2 expression on the tumor vasculature further providing a mechanism for the anti-angiogenic activity of these compounds. Overall, these results suggest that inhibition of angiogenesis is the main mechanism by which AIs suppress growth of benign and malignant lesions in this model of NSCLC.

Tumor associated macrophages (TAMs) are a key component of tumor microenvironment and have been implicated in tumor progression and angiogenesis. It has been shown that NSCLC patients with higher density of TAMs have lower median relapse-free survival (7 months) compared to patients whose tumors had lower density of TIMs (26 months survival) [18]. Macrophage staining (using F4-80) indicated infiltration of these TAMs in the lung in vehicle treated mice (Figure 4). Treatment with AIs particularly sunitinib and axitinib was associated with lower density of TAMs further suggesting an additional mechanism for anti-tumor efficacy of AIs in Kras^G12D-LSL^ lung tumors.

## Discussion

This study reports anti-tumor efficacy of three different RTKIs including PF-210, axitinib and sunitinib in spontaneous tumors in lung in Kras^G12D-LSL^ GEMMs. The high failure rate of clinical trials in late stage cancer patients warrants development of mouse tumor models which are more relevant to the human diseases. GEMMs, carrying genetic alterations similar to what is observed in cancer patients, might represent a more relevant tumor model to predict clinical outcome.

The VEGF signaling pathway is one of the major signaling pathways in tumor angiogenesis in many cancers. An anti-VEGF monoclonal antibody, bevacizumab, has been approved in combination with chemotherapy for the treatment of NSCLC [19]. Bevacizumab is the first targeted agent to improve survival in advanced-stage NSCLC patients when combined with first-line chemotherapy. In the present study, we use sunitinib, axitinib, PF-210 all of which targeting VEGFR signaling pathway with different pharmacokinetic and pharmacodynamic properties [7,20]. Our results show that reduction of malignant lesions in lungs is the common and consistent theme among all the above compounds. Progression of malignant lesions prior to diagnosis and treatment are the major contributors to low survival rate in NSCLC patients [18]. Lack of efficacy of these agents in hyperplastic lesions indicate that angiogenesis may not play a significant role in growth of pre-neoplastic lesions lung tumors in Kras^G12D-LSL^ GEMMs. Additionally while sunitinib is a multi-targeting RTKIs, our data indicate that, at clinical dose, targeting PDGFR-β, KIT and CSF1-R does not provide additional efficacy compared to PF-210 and axitinib which are selective inhibitors of VEGF. These data once again signifies the role of VEGF as a key regulator of tumor angiogenesis in a preclinical model of NSCLC. PF-210 showed superior efficacy in suppressing benign neoplasia lesions compared to axitinib and sunitinib. Future investigations might provide some insight into the mechanism of action of PF-210.

Histopathological analysis showed that all these AIs target tumor vasculature to inhibit growth of malignant lesions. Moreover, most of the tumor blood vessels in treated mice lacked smooth muscle cell coverage suggesting a role for VEGF in establishment of a cross talk between smooth muscle cells and endothelial cells. Furthermore, AI- treated mice had lower number of TAMs compared to the vehicle treated animals suggesting that these cells may play a proangiogenic role in this model [21]. Future studies will determine if AIs alter homing of macrophages to the tumors or are directly targeting them. In addition, further investigation is warranted to understand pharmacokinetics and pharmacodynamics of these compounds in the tumors which may describe differences in the mechanism of action of AIs in the current study.

## Conclusion

Our data indicate that small molecule inhibitors of VEGF pathway suppress growth of adenocarcinoma lesions in a NSCLC model of Kras^G12D-LSL^ GEMM by targeting components of tumor vasculature and stroma.

## Competing interests

All authors are employees and are stock holders of Pfizer Incorporations.

## Authors’ contributions

FS designed all the studies and experiments, BKM performed all the histology staining and analyzed the data, JHL, BHS and FS executed in vivo study, BKM and FS wrote the manuscript. All authors read and approved the final manuscript.

## Pre-publication history

The pre-publication history for this paper can be accessed here:

http://www.biomedcentral.com/1471-2407/13/213/prepub
